# Understanding the roles of salt-inducible kinases in cardiometabolic disease

**DOI:** 10.3389/fphys.2024.1426244

**Published:** 2024-07-16

**Authors:** Fubiao Shi

**Affiliations:** Department of Medicine, Division of Cardiovascular Medicine, Vanderbilt University Medical Center, Nashville, TN, United States

**Keywords:** salt-inducible kinases, cardiovascular disease, metabolic syndrome, inflammation, fibrosis, SIK inhibitors

## Abstract

Salt-inducible kinases (SIKs) are serine/threonine kinases of the adenosine monophosphate-activated protein kinase family. Acting as mediators of a broad array of neuronal and hormonal signaling pathways, SIKs play diverse roles in many physiological and pathological processes. Phosphorylation by the upstream kinase liver kinase B1 is required for SIK activation, while phosphorylation by protein kinase A induces the binding of 14-3-3 protein and leads to SIK inhibition. SIKs are subjected to auto-phosphorylation regulation and their activity can also be modulated by Ca^2+^/calmodulin-dependent protein kinase in response to cellular calcium influx. SIKs regulate the physiological processes through direct phosphorylation on various substrates, which include class IIa histone deacetylases, cAMP-regulated transcriptional coactivators, phosphatase methylesterase-1, among others. Accumulative body of studies have demonstrated that SIKs are important regulators of the cardiovascular system, including early works establishing their roles in sodium sensing and vascular homeostasis and recent progress in pulmonary arterial hypertension and pathological cardiac remodeling. SIKs also regulate inflammation, fibrosis, and metabolic homeostasis, which are essential pathological underpinnings of cardiovascular disease. The development of small molecule SIK inhibitors provides the translational opportunity to explore their potential as therapeutic targets for treating cardiometabolic disease in the future.

## Introduction

Salt-inducible kinases (SIKs), including SIK1, SIK2, and SIK3, are serine/threonine kinases of the adenosine monophosphate-activated protein kinase (AMPK) family (Jagannath et al., 2023). The founding member SIK1 was first described as a myocardial sucrose-nonfermenting 1 (SNF1)-like kinase (*msk*) involving mouse heart development (Ruiz et al., 1994). It was cloned later as a protein kinase of 776 amino acids from rats adrenal gland after high-salt diet treatment (Wang et al., 1999), thereby the name of *salt-inducible kinase* was first devised. The two additional members SIK2 and SIK3 were identified later through *in silicon* studies based on protein sequence similarity (Horike et al., 2003; Katoh et al., 2004a).

The three SIK isoforms are broadly expressed in vertebrate tissues. Based on the transcriptomic data from human and mouse tissues, SIK1, SIK2, and SIK3 are mostly enriched in the adrenal gland, the adipose tissue, and the brain, respectively (Fagerberg et al., 2014; Yue et al., 2014). SIK activities are dynamically regulated by multiple physiological cues through both transcriptional and post-translational mechanisms. SIK1 expression is regulated by cyclic AMP (cAMP) through the action of the cAMP responsive element binding (CREB) protein (Jagannath et al., 2013). The mRNA expression of SIK1 can be induced by many physiological cues, such as high-salt dietary intake (Wang et al., 1999), membrane depolarization (Feldman et al., 2000), adrenocorticotropic hormone (Lin et al., 2001), fasting (Koo et al., 2005), adrenergic stimulations (Kanyo et al., 2009), transforming growth factor β (TGFβ) signaling (Lönn et al., 2012), circadian clock (Jagannath et al., 2013), and hypertrophic cardiac stresses (Hsu et al., 2020). In contrast, SIK2 and SIK3 are constitutively expressed but their expression can be modulated under certain pathophysiological conditions. For example, SIK2 and SIK3 mRNA expression are downregulated in the adipose tissue of diabetic and obese individuals (Säll et al., 2016).

**FIGURE 1:**
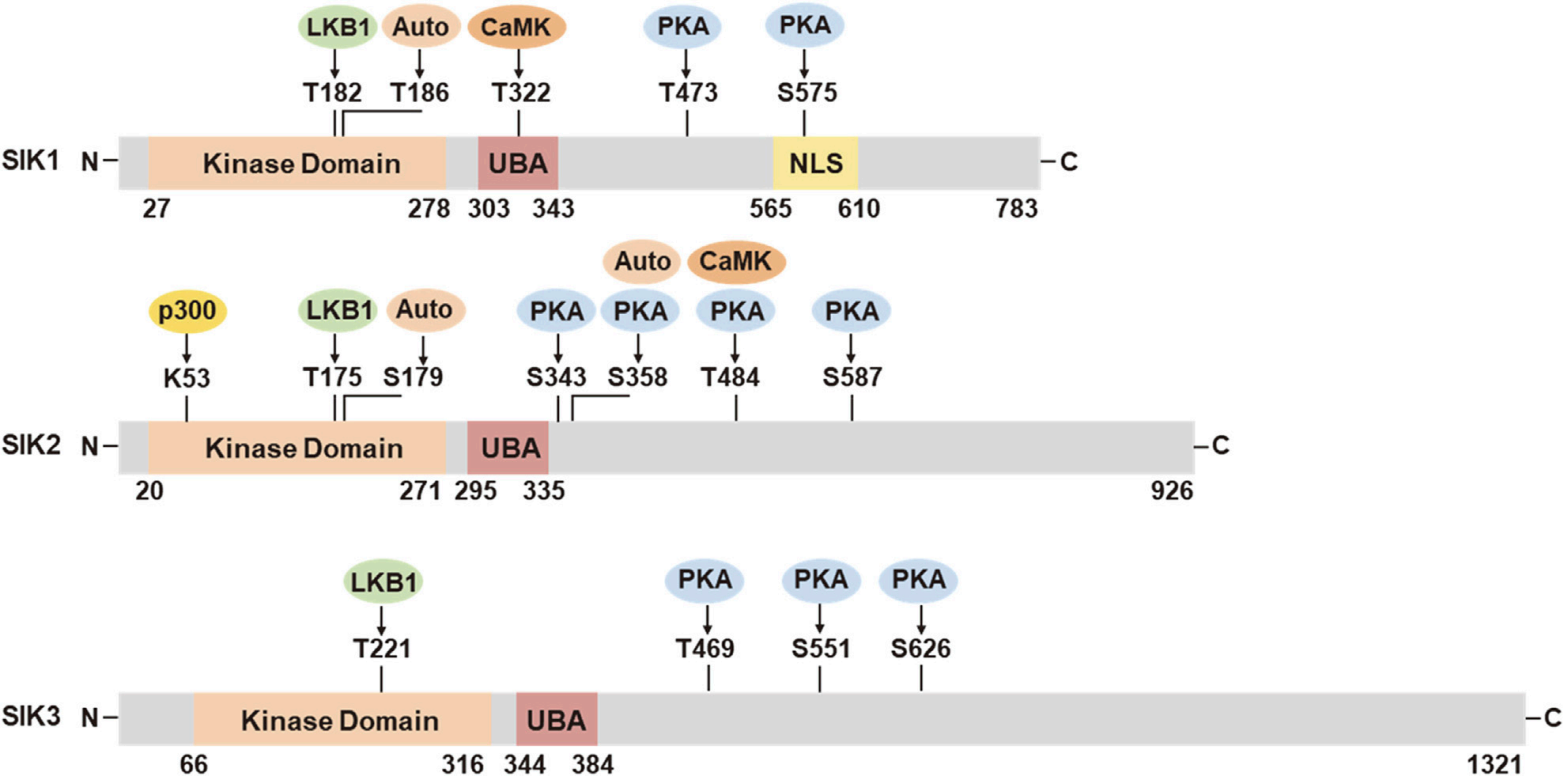
Functional domains and post-translational modifications in SIK1, SIK2, and SIK3. UBA, ubiquitin associated domain; NLS, nuclear location sequences; LKB1, liver kinase B1; Auto, autocatalysis; PKA, protein kinase A; CaMK, Ca^2+^/calmodulin dependent kinase; p300, histone acetyltransferase. The numbers of amino acid residues refer to human proteins SIK1 (NP_775490.2), SIK2 (NP_056006.1), and SIK3 (NP_079440.3), respectively.

SIK proteins process an N-terminal serine/threonine kinase domain (KD), followed by a ubiquitin-associated (UBA) domain, and a C-terminal domain (CTD) (Figure 1). The KD is well-conserved among the three isoforms. It contains a liver kinase B1 (LKB1) phosphorylation threonine site in the activation loop (T-loop). The UBA domain is located within a SNF1 homolog (SNH) region following the KD. The regulatory CTD varies in length among the three isoforms and contains multiple protein kinase A (PKA) phosphorylation sites. SIK1 also has nuclear localization sequences (NLS) (Katoh et al., 2002) and it can repress CREB activity both in the nucleus and cytoplasm (Katoh et al., 2004b).

SIK activities are tightly regulated through post-translational modification in response to hormonal stimulation and physiological alternations (Figure 1). The activation of SIK kinase activity requires the phosphorylation by LKB1 in the activation loop near the substrate binding sites (SIK1 Thr182, SIK2 Thr175, and SIK3 Thr221). The UBA domain assists the LKB1 phosphorylation and SIK kinase activation, potentially by preventing the binding of SIKs with the 14-3-3 phospho-binding proteins (Al-Hakim et al., 2005; Jaleel et al., 2006). Activation by LKB1 stimulates the autophosphorylation in the activation loop of SIK1 (Ser186) and SIK2 (Ser179), which are important for sustained kinase activity (Hashimoto et al., 2008). SIK2 can also be auto-phosphorylated at Ser358, which regulates it protein stability (Henriksson et al., 2012). SIK1 and SIK2 can be phosphorylated by Ca^2+^/calmodulin dependent kinase (CaMK). Intracellular sodium-mediated SIK1 activation requires its phosphorylation by CaMK I at Thr322 (Sjöström et al., 2007; Bertorello and Zhu, 2009). Phosphorylation of SIK2 by CaMK I/V at Ser484 promotes its protein degradation (Sasaki et al., 2011). PKA phosphorylation sites have been identified in SIK1 (Thr473, Ser575), SIK2 (Ser343, Ser358, Thr484, Ser587), and SIK3 (Thr469, Ser551, Ser626) (Sonntag et al., 2018). PKA phosphorylation facilitates the binding of 14-3-3 protein and leads to SIK inhibition. PKA-mediated SIK inhibition is underpinning a variety of neuronal and hormonal factor actions, such as glucagon, prostaglandins, parathyroid hormone, and α-melanocyte stimulation hormone (Sakamoto et al., 2018; Wein et al., 2018). SIKs are also subjected to additional post-translational modification (Sun et al., 2020), such as histone acetyltransferase (HAT) p300-mediated inhibitory acetylation and HDAC6-mediated deacetylation in SIK2 at Lys53 (K53) (Bricambert et al., 2010; Yang et al., 2013), and E3 ligase RNF2-mediated ubiquitination in SIK1 (Qu and Qu, 2017).

SIKs regulates physiological processes through direct phosphorylation of their substrates. The SIK phosphorylation sites have been defined through *in vitro* studies as a motif of LXB(S/T)XS*XXXL (B, basic amino acid; X, any amino acid) (Screaton et al., 2004). SIK phosphorylation leads to 14-3-3 binding and cytoplasmic retention of their substrates. Following PKA-mediated SIK inhibition, SIK substrates will become dephosphorylated and translocated to the nucleus to control the target gene program. The most well-characterized SIK substrates include the class IIa histone deacetylases (HDACs, HDAC4, 5, 7 and 9) and the CREB-regulated transcriptional coactivators (CRTCs, CRTC1, 2 and 3). Despite their name, the histone deacetylation activity of class IIa HDACs towards histones are relatively weak. This is due to the differences in amino acid composition in their active site as compared to the *bona fide* HDACs (Asfaha et al., 2019; Park and Kim, 2020). Instead, they can interact and remove the acetylation marks in a variety of non-histone proteins or transcriptional coregulators and hereby control the transcriptional activity of these targets. In addition, class IIa HDACs may associate with HDAC3 (a member of the class I HDACs that possesses *bona fide* histone deacetylation activity) and nuclear co-repressors and transcription factors NCoR/SMRT as an active complex that can drive epigenetic changes (Fischle et al., 2002). In their dephosphorylated states, CRTCs are translocated to the nucleus and interact with CREB via the basic leucine zipper (bZIP) domain to promote the CREB-dependent transcriptional gene program (Altarejos and Montminy, 2011). Mechanistically, the interaction with CRTCs increase the stability of CREB and binding to target gene promoters (Katoh et al., 2006). CRTCs also facilitate the recruitment of HAT p300 for CREB transcription activity (Altarejos and Montminy, 2011).

In response to external hormonal and neuronal factor stimulation, the second messenger cAMP signaling stimulates PKA-mediated phosphorylation and leads to SIK inhibition. This will release the cytosolic retention of SIK substrates and facilitate their nuclear translocation to control target gene program. Class IIa HDACs functions as a potent suppressor of the myocyte enhancer factor 2 (MEF2)-dependent gene program, which controls a variety of pathological and developmental processes, including cardiac hypertrophy (Zhang et al., 2002), muscle development and fiber-type formation (Edmondson et al., 1994; Handschin et al., 2003), myocyte survival and growth (Shen et al., 2006; Berdeaux et al., 2007; Stewart et al., 2012), craniofacial development (Verzi et al., 2007), and bone formation (Arnold et al., 2007; Collette et al., 2012). CRTCs functions as a coactivator to promote the CREB-dependent gene program, which involves in many metabolic pathways, such as the glucose uptake (Qi et al., 2009; Weems et al., 2012; Henriksson et al., 2015; Nixon et al., 2015), lipogenesis (Yoon et al., 2009; Bricambert et al., 2010; Park et al., 2014), gluconeogenesis (Patel et al., 2014; Itoh et al., 2015), steroidogenesis (Doi et al., 2002; Lee et al., 2016), and inflammatory cytokine production (Clark et al., 2012; MacKenzie et al., 2012). Furthermore, the transcription of *Sik1* mRNA is regulated in a CREB-dependent manner (Jagannath et al., 2013), suggesting a negative feedback regulation between SIK1 and the CRTCs-CREB pathway.

Although HDACs and CRTCs are well-described SIK substrates, additional substrates have been identified. SIK1 can phosphorylate phosphatase methylesterase-1 (PME-1) of the protein phosphatase 2A (PP2A) complex to activate the activity of Na^+^/K^+^-ATPase (NKA) (Sjöström et al., 2007) (more details in next section). SIK1 functions downstream of TGFβ to negatively regulate type I receptor kinase signaling (Kowanetz et al., 2008; Lönn et al., 2012). Mechanistically, SIK1 forms a complex with SMAD7 (mothers against decapentaplegic homolog 7) and the E3 ubiquitin-protein ligase SMURF2 (SMAD-specific E3 ubiquitin-protein ligase 2) to promotes protein degradation of the activated type-I TGFβ receptor (TGFBR1 or ALK5) (Kowanetz et al., 2008; Lönn et al., 2012). SIK1 can phosphorylate the polarity protein PAR3 (Partitioning defective 3) to regulate tight junction assembly (Vanlandewijck et al., 2017). SIK1 can phosphorylate and activate the nucleotide release channel Pannexin 1 (PANX1) at Ser205, which involves the inflammatory responses of T cell to restrict the severity of allergic airway inflammation (Medina et al., 2021). In addition, SIK2 can form a complex with the cyclin-dependent kinase 5 (CDK5) regulatory subunit 1 (CDK5R1, also known as p35) and the E3 ligase PJA2 (praja ring finger ubiquitin ligase 2) to promotes insulin secretion (Sakamaki et al., 2014).

**FIGURE 2:**
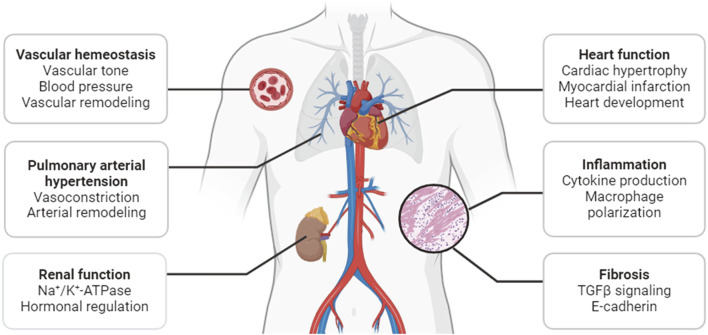
Functions of SIKs in the cardiovascular system.

Acting as mediators of a broad array of neuronal and hormonal signaling pathways, SIKs play diverse roles in many physiological and pathological processes. Earlier studies and recent progress have demonstrated that they are critical regulators of the pathophysiological processes in cardiovascular and metabolic disease. This review will summarize our current understanding of the cardiovascular function of SIKs, with a focus on their pathophysiological roles in sodium handling, vascular remodeling, pulmonary arterial hypertension, cardiac hypertrophy and ischemia, inflammation, and fibrosis (Figure 2).

## Sodium sensing and salt intake

The Na^+^/K^+^-ATPase (NKA) is a sodium/potassium-transporting ATPase wildly expressed on the outer plasma membrane. For each ATP consumed per transport cycle, NKA transports 3 sodium out of the cell and 2 potassium into the cell (Pirahanchi et al., 2024). Structurally, the NKA is composed of three subunits assembled in a 1:1:1 stoichiometry–the catalytic alpha (α)-subunit, the extracellular beta (β)-subunit, and the regulatory FXYD subunit (Nguyen et al., 2022). NKA activity helps to maintain the ion concentration gradient and cellular membrane potential, hereby the sodium and potassium gradients across the plasma membrane can be utilized by animal cells in various physiological processes for specialized functions (Jaitovich and Bertorello, 2010). As such, the range of the gradient demands requires NKA activity to be fine-tuned to coordinate different cellular needs (Jaitovich and Bertorello, 2010).

**FIGURE 3:**
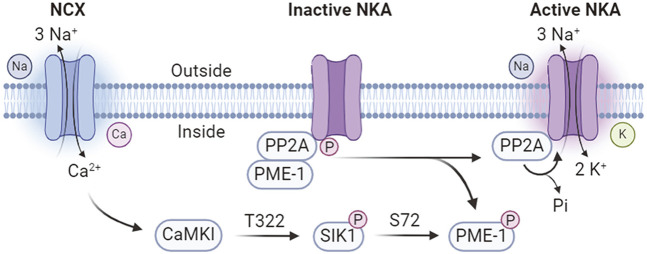
Calcium-dependent SIK1 regulation of NKA activity. NCX, Na^+^/Ca2^+^ exchanger; NKA, Na^+^/K^+^-ATPase; PP2A, protein phosphatase 2A; PME-1, phosphatase methylesterase-1; CaMKI, Ca2+/calmodulin dependent kinase I.

SIK1 involves in an intracellular sodium sensing network that controls NKA activity through a calcium-dependent process (Sjöström et al., 2007) (Figure 3). SIK1 was associated with NKA in the renal epithelium cells and participates in the regulation of NKA activity in response to sodium changes (Sjöström et al., 2007). Increases in intracellular sodium are coupled with calcium influx through the reversible Na^+^/Ca2^+^ exchanger (NCX), which is co-localized with NKA (Moore et al., 1993; Dostanic et al., 2004; Iwamoto et al., 2004). The sodium-induced calcium influx leads to the activation of SIK1 by CaMK-dependent phosphorylation at Thr322 (Sjöström et al., 2007). In its inactive form, the NKA catalytic α-subunit is constitutively associates with the protein phosphatase 2A (PP2A)/phosphatase methylesterase-1 (PME-1) complex (Sjöström et al., 2007). Activated SIK1 phosphorylates PME-1 possibly at Ser72 (Jaitovich and Bertorello, 2010), leading to its dissociation from the NKA/PP2A complex and allowing the dephosphorylation of NKA by PP2A to attain its catalytic activity (Sjöström et al., 2007). In addition, NKA activity can be regulated at the gene expression levels (Taub, 2018). The expression of *ATP1B1* gene, encoding the NKA β1-subunit, is transcriptionally regulated by CRTCs (Taub, 2018). SIKs promote the cytoplasmic retention of CRTCs and hereby negatively regulates *ATP1B1* expression (Taub et al., 2010). Extracellular stimulations by renal effectors such as norepinephrine, dopamine, and prostaglandins leads to the inhibition of SIK1 and nuclear translocation of CRTCs to promote *ATP1B1* transcription via CREB (Taub et al., 2015). Furthermore, SIK1 also involves in sodium-independent hormonal regulation of NKA activity such as the dopamine and angiotensin pathways to control blood pressure homeostasis (Jaitovich and Bertorello, 2010).

Genetic knockout mouse models have demonstrated the important roles of SIK1 in the pathological vascular adaptations to high-salt (HS) intake. HS intake induces *Sik1* expression in the vascular smooth muscle cell (VSMCs) layer of the aorta (Bertorello et al., 2015). Under normal salt condition, *Sik1* deletion does not affect blood pressure (BP) but increase collagen deposition in the aorta (Bertorello et al., 2015). On HS condition, *Sik1* deletion leads to increased BP and vascular remodeling, as indicated by higher systolic BP, upregulation of TGFβ1 signaling, increased expression of endothelin-1 and genes related to VSMC contraction, and signs of cardiac hypertrophy (Bertorello et al., 2015). In *in vitro* cell models, *Sik1* knockdown increases collagen in aortic adventitial fibroblasts and enhances the expression of endothelin-1 and contractile markers in VSMCs (Bertorello et al., 2015). These data suggested that vascular SIK1 prevents salt-induced hypertension by regulating vasoconstriction via downregulation of TGFβ1 signaling (Bertorello et al., 2015). Furter study showed that HS intake induced hypertension in *Sik1* ^*−/−*^ mice is associated with overactivity of the sympathetic nervous system (SNS), as indicated by increased levels of urinary L-3,4-dihydroxyphenylalanine (L-DOPA) and noradrenaline, and higher adrenal dopamine β-hydroxylase (DβH) activity (Pires et al., 2019). Preadministration of etamicastat, a peripheral reversible DβH inhibitor, prevents the HS-induced systolic BP increase, suggesting that SNS overactivity is a key mediator of salt-induced hypertension in *Sik1* ^*−/−*^ mice (Pires et al., 2019).

SIK1 and SIK2 play distinct roles in mediating the HS-induced high BP and cardiac hypertrophy in mouse models. *Sik2* deletion does not affect the BP but prevents the development of left ventricular hypertrophy (LVH) on chronic HS intake (Popov et al., 2014). Instead, combined *Sik1/2* deletion leads to higher BP but does not alter LVH on chronic HS intake, suggesting that SIK1 is required for maintaining normal BP while SIK2 is required for HS-induced cardiac hypertrophy independent of high BP (Pires et al., 2021). Further evidence also supports a pathological role for SIK2 in cardiac hypertrophy. For example, the hypertensive variant of α-adducin is associated with higher *Sik2* expression and LVH in hypertensive Milan rats (Popov et al., 2014). In addition, the mRNA expression of *SIK2*, α-adducin, and genes related to cardiac hypertrophy are also positively correlated in human cardiac biopsies (Popov et al., 2014).

## Vascular remodeling

The blood vessel is an autocrine-paracrine complex composed of VSMCs, endothelium cells, and fibroblasts (Gibbons and Dzau, 1994). In response to the hemodynamic alterations, the blood vessels undergo a structural remodeling processes involving in cell growth, death, migration, and production or degradation of the extracellular matrix (ECM) (Gibbons and Dzau, 1994). SIK1 plays an important role in maintaining vascular homeostasis. SIK1 is localized in human VSMCs and endothelial cells (Popov et al., 2011). Lower SIK1 activity leads to decreased NKA activity in VSMCs and is associated with increased vascular tone (Popov et al., 2011; Bertorello et al., 2015). A polymorphism in SIK1 gene encoding a Gly15 to Ser (G15S) missense mutation increases plasma membrane NKA activity in cultured VSMCs and is associated with lower blood pressure and reduced left ventricle (LV) mass in two separate Swedish and Japanese cohorts (Popov et al., 2011). In addition, SIK1 also involves in the negative feedback regulation of the TGFβ1 signaling and SIK1 deletion increases the expression of genes related to ECM remodeling (Bertorello et al., 2015). Furthermore, SIK1 regulates the expression of E-cadherin and contribute to LKB1-mediated regulation of cell polarity and intercellular junction stability (Eneling et al., 2012).

SIKs have been implicated in the pathological vascular remodeling process. Vascular calcification is a pathologic osteochondrogenic process of the blood vessels and is widely used as a clinical indicator of atherosclerosis (Abedin et al., 2004). SIKs promote vascular calcification by inducing cytoplasmic retention of HDAC4 (Abend et al., 2017). HDAC4 is induced in early calcification process, and it acts with the cytoplasmic adaptor protein ENIGMA (Pdlim7) to promote the VSMC calcification phenotypes (Abend et al., 2017). Pharmacologic SIK inhibition facilitates HDAC4 nuclear translocation and leads to the suppression of calcification process (Abend et al., 2017). Arterial restenosis is a pathological arterial narrowing process that results from a combination of elastic recoil, thrombosis, remodeling and intimal hyperplasia (Kenagy, 2011). SIK3 promotes arterial restenosis by stimulating VSMC proliferation and neointima formation (Cai et al., 2021). SIK3 is highly expressed in proliferating VSMCs and is also highly induced by growth medium *in vitro* and in neointimal lesions *in vivo* (Cai et al., 2021). Inactivation of SIKs attenuates the proliferation and migration of VSMCs, and reduced neointima formation and vascular inflammation in a femoral artery wire injury model (Cai et al., 2021).

## Pulmonary arterial hypertension

Pulmonary arterial hypertension (PAH) is a high blood pressure condition in the arteries of lung. The pathogenesis of PAH involves in progressive narrowing of the arteries due to vasoconstriction, thrombosis, and vascular remodeling (Lan et al., 2018). Narrowing of the pulmonary arteries induces higher flow resistance and arterial pressure which cause right ventricle (RV) overload and ultimately lead to RV failure (António et al., 2022). Vascular remodeling is a hallmark feature in PAH and is characterized by excessive cell proliferation, abnormal release of inflammatory cytokines such as interleukin 1 (IL-1), IL-6, and tumor necrosis factor alpha (TNFα), and the upregulation of growth factors such as TGFβ (António et al., 2022). PAH is a multifactorial disease. The familiar form of PAH has been linked with mutations in genes of the TGFβ signaling pathway (Liu et al., 2011; Reynolds et al., 2011; Frydman et al., 2012) and mutations in ion channels such as KCNK3 (K^+^ channel subfamily K member 3) (Ma et al., 2013) and ATP1A2 (NKA α2-subunit) (Montani et al., 2013). As SIKs coordinate signaling pathways implicated in vasoconstriction, inflammation, and vascular remodeling, it has been proposed that the dysregulation of SIK pathways might underpin the pathophysiology of PAH (António et al., 2022).

Aberrant proliferation of the pulmonary artery endothelial cells (PAECs) plays an important role in the pathogenesis of PAH. It has been shown that the transcriptional factor MEF2 regulates expression of a number of transcriptional targets involved in pulmonary vascular homeostasis (Kim et al., 2014). The activity of MEF2 is significantly impaired in the PAECs derived from subjects with PAH due to the excess nuclear accumulation of HDAC4 and HDAC5 (Kim et al., 2014). Pharmacological inhibition by a selective class IIa HDACs inhibitor MC1568 restores MEF2 activity and transcriptional target expression, suppresses cell migration and proliferation, and rescues pulmonary hypertension in experimental mouse models (Kim et al., 2014). As SIKs are known to inhibit type IIa HDACs and able to restore MEF2-mediated transcription, it has been proposed that maneuvers that upregulate SIK activity could offer new opportunity to explore disease-modifying strategy for PAH (António et al., 2022).

Arterial remodeling in PAH also involves in the excessive proliferation and migration of pulmonary artery smooth muscle cells (PASMCs). A recent study showed that SIK1 deficiency stimulates PASMC proliferation via upregulation of the yes-associated protein (YAP) pathway and promotes vascular remodeling in PAH (Pu et al., 2022). The expression of SIK1, but not SIK2 or SIK3, is suppressed in the lung tissues of experimental PAH mice and in cultured human PASMCs under hypoxia (Pu et al., 2022). Pharmacological SIK inhibition or AAV9-mediated *Sik1* knockdown in smooth muscles aggravates RV hypertrophy and pulmonary arterial remodeling in a hypoxia-induced PAH mouse model (Pu et al., 2022). *Sik1* knockdown or inhibition promote the nuclear accumulation of YAP and stimulate proliferation and migration of human PASMCs under hypoxia condition (Pu et al., 2022). This study supported an anti-proliferative role for SIK1 in hypoxia-induced PAH remodeling and suggested that SIK1 might be a potential target for the treatment of PAH (Pu et al., 2022). However, the pathological role for SIK1 in alternative PAH models and in PAH patients is yet to be further explored.

## Cardiac hypertrophy and ischemia

SIK1 was originally identifies as a myocardial SNF1-like kinase (*msk*) in a screen for novel protein kinases involving mouse heart development (Ruiz et al., 1994). *Sik1* mRNA expression is restricted in the myocardial progenitor cells and marks the promotive ventricle and atrium in the developing heart (Ruiz et al., 1994), suggesting a possible role for SIK1 in embryonic myocardial cell differentiation and primitive heart formation (Ruiz et al., 1994). A later study further showed that *Sik1* deletion in mouse embryonic stem cells postpones cardiomyocyte differentiation possibly by downregulation of the cyclin-dependent kinase inhibitor protein (Romito et al., 2010).

SIKs involve in high-salt (HS)-induced cardiac hypertrophy. HS intake is associated with cardiac hypertrophy both in humans (Du Cailar et al., 1992; Frohlich and Varagic, 2004) and animal models (Ferreira et al., 2010). A HS diet is associated with hallmark features of pathological cardiac remodeling, such as alterations in myocardial mechanical performance, dysregulation of the α/β-myosin heavy chain (MHC) expression, and alterations in calcium homeostasis and myocardial contractility (Patel and Joseph, 2020). Increases in intracellular sodium activates SIK1 and increases MEF2 transcriptional activity and cardiac hypertrophy signature gene expression in myocardial cells (Popov et al., 2012). The salt-induced activation of SIK1 and MEF2 is coupled by intracellular calcium influx through the NCX and activation of CaMKI (Popov et al., 2012). In addition, systemic hypertension also leads to a shift in the isoform distribution towards a decrease in the α/β-MHC ratio (Patel and Joseph, 2020). Cardiac hypertrophy of the spontaneous hypertensive rats is associated with decreased cardiac expression of SIK1 and SIK3 (Pinho et al., 2012). In contrast, SIK2 activation has been implicated in cardiac hypertrophy. SIK2 deletion prevents HS-induced cardiac hypertrophy independent of high BP (Pires et al., 2021). In hypertensive Milan rats, the hypertensive variant of α-adducin is associated with higher *Sik2* expression and LVH (Popov et al., 2014). Furthermore, the mRNA expression of *SIK2*, α-adducin, and genes related to cardiac hypertrophy are positively correlated in human cardiac biopsies (Popov et al., 2014).

**FIGURE 4:**
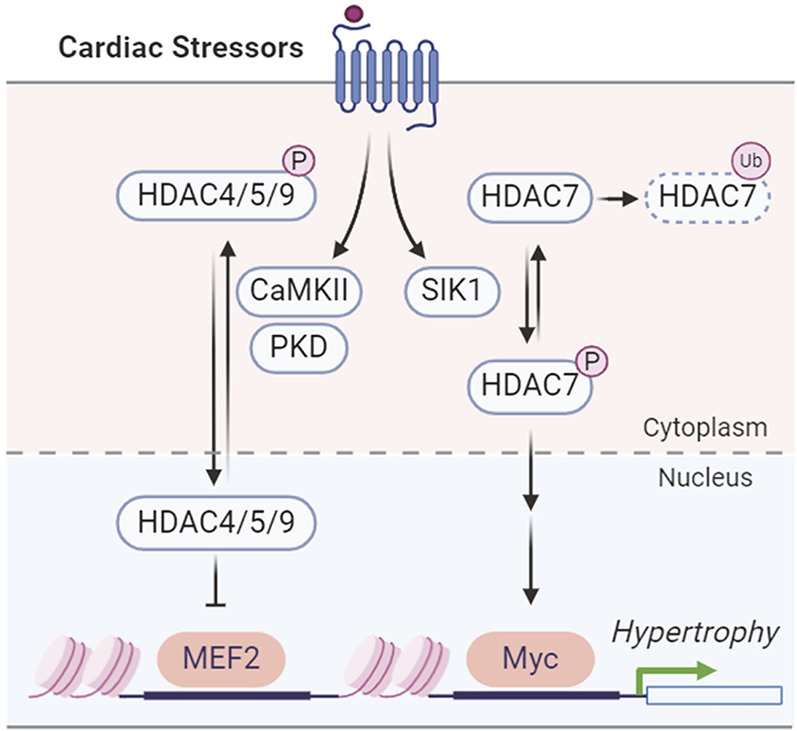
SIK1 and CaMKII/PKD regulation of HDACs in cardiac hypertrophy.

SIKs have been implicated in pressure overload-induced hypertrophic cardiac remodeling. In adult mice, *Sik1* deletion protects against hypertrophic cardiac remodeling and heart failure following pressure overload via transverse aortic constriction (Hsu et al., 2020). Mechanistically, SIK1 phosphorylates and stabilizes HDAC7 to increase the expression of c-Myc, which in turn promotes the pro-hypertrophic gene program (Hsu et al., 2020) (Figure 4). In contrast, other class IIa HDACs, including HDAC4, HDAC5, and HDAC9, are regulated by stress-induced CaMKII and protein kinase D (PKD) phosphorylation for nuclear export in cardiomyocytes, which will de-suppress MEF2 to stimulate pro-hypertrophic gene expression (Zhang et al., 2002; Chang et al., 2004; Lehmann et al., 2017; Travers et al., 2020) (Figure 4). This suggests the functional divergence of HDAC7 from the other class IIa HDACs in cardiomyocyte remodeling (Hsu et al., 2020; Travers et al., 2020). A recent study further reported that the SIK1 expression is potentially regulated by the master circadian rhythm factor BMAL1 and *Sik1* knockdown in neonatal rat ventricular myocytes (NRVMs) reduces myocyte size and hypertrophic gene expression in response to phenylephrine stimulation (Arrieta et al., 2023). Unlike the pro-hypertrophic role of SIK1, SIK3 functions as a suppressor of cardiac hypertrophy (Hsu, 2022). Loss of SIK3 induces hypertrophic phenotypes in cultured NRVMs and in adult mouse hearts (Hsu, 2022). Mechanically, SIK3 deletion reduces ARHGAP21 (Rho GTPase activating protein 21) protein and promotes the nuclear translocation of MRTF-A (myocardin related transcription factor A) to stimulate hypertrophic gene program (Hsu, 2022).

SIKs also involve in the pathological cardiac remodeling following myocardial infarction (MI). A recent study reported that intramyocardial transplantation of embryonic cardiopulmonary progenitors can target the SIK1-CREB1 axis via exosomal transfer of miR-27b-3p to facilitate cardiac repair and improve cardiac function in a mouse model of MI (Xiao et al., 2024). Ischemia-reperfusion (IR) injury induces the expression of SIK2 in myocardial tissues (Liu et al., 2022). Overexpression of SIK2 in NRVMs increases AKT phosphorylation but suppresses the activity of YAP, one of the downstream effectors of Hippo pathway (Facchi et al., 2022). However, SIK2 overexpression does not impact NRVM proliferation or survival, but leads to an increase in cardiomyocyte hypertrophy (Facchi et al., 2022). *Sik2* deletion suppresses hypertrophic response and reduces infarct area and cardiac fibrosis in MI induced by coronary artery ligation, suggesting that SIK2 deficiency protects against cardiac ischemia (Facchi et al., 2022). However, the detailed mechanisms by which SIK2 promotes the hypertrophic gene program is yet to be revealed.

## Inflammation

Inflammation is a coordinated immune response of the body to defense against injuries, infection, and stress (Pahwa et al., 2024). It is an essential pathological component closely associated with cardiovascular disease. For example, atherogenesis is a process associated with endothelial cell dysfunction that leads to atherosclerotic plaque formation and coronary arterial heart disease (Alfaddagh et al., 2020). It is considered that these pathological processes are driven by the cytokines, interleukins (ILs), and cellular constituents of the inflammatory response (Alfaddagh et al., 2020). Pathological cardiac remodeling is a process involving cardiac myocyte growth and death, vascular rarefaction, fibrosis, inflammation, and electrophysiological remodeling (Burchfield et al., 2013). There are growing evidences showing that elevated inflammatory biomarkers, implicating innate immune cells such as macrophages, are associated with worsened clinical outcomes in heart failures (HF) (Kalogeropoulos et al., 2010; Chen and Frangogiannis, 2016; Hage et al., 2017; DeBerge et al., 2019).

**FIGURE 5:**
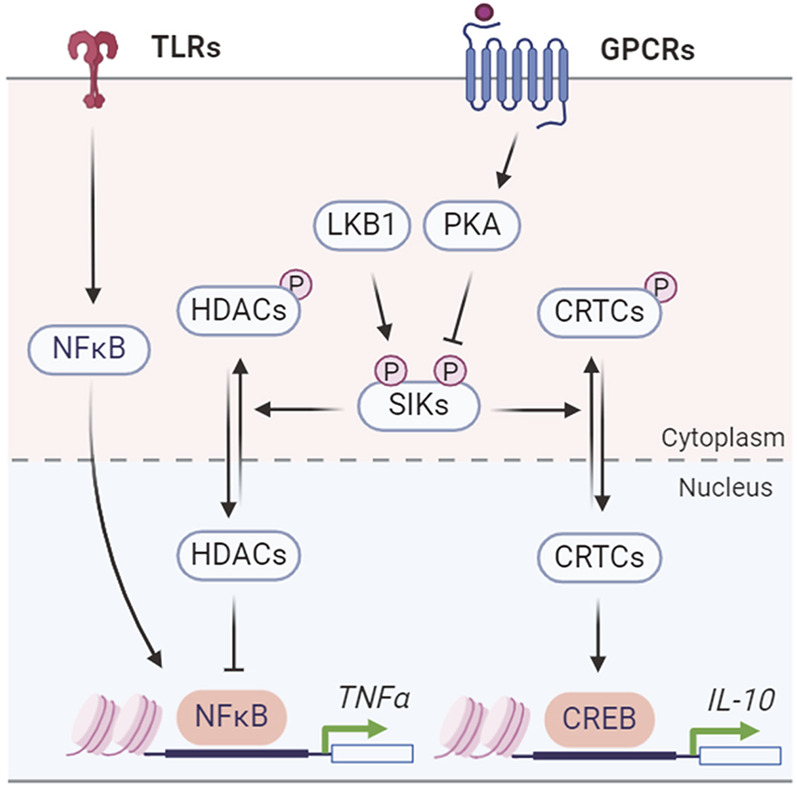
SIK regulation of inflammatory cytokine production.

SIKs regulate the production of inflammatory cytokines, such as TNFα and ILs, to coordinate the innate immune responses (Figure 5). The endogenous SIK activity suppresses the anti-inflammatory signaling in macrophages. Pharmacological SIK inhibition increases secretion of anti-inflammatory cytokine IL-10 but suppresses proinflammatory cytokines TNFα, IL-6, and IL-12 in macrophage after toll-like receptor (TLR) stimulation by lipopolysaccharide (LPS) (Clark et al., 2012). The anti-inflammatory IL-10 expression depends on the CRTC3-CREB transcription activity (Clark et al., 2012), while the proinflammatory cytokine expression depends on HDAC4-mediated regulation of the nuclear factor-κB (NF-κB) pathway (Luan et al., 2014). SIKs mediate the prostaglandin E2 (PGE_2_) signaling to promote IL-10 production and macrophage polarization (MacKenzie et al., 2012). Combined stimulation with PGE_2_ and LPS leads to IL-10 expression and an anti-inflammatory phenotype in macrophages (Darling et al., 2016). Similarly, PGE_2_ stimulates IL-10 mRNA expression through CRTC3-CREB-mediated transcription and either genetic or pharmacological SIK inhibition mimics the effect of PGE_2_ on IL-10 production (Darling et al., 2016). Characterization of the primary macrophages carrying a catalytically inactive mutation in individual SIK gene further revealed that all three SIK isoforms contribute to macrophage polarization, with a major role for SIK2 and SIK3 (Darling et al., 2016). However, conflicting results have shown that SIKs act to suppress the pro-inflammatory signaling in Raw 264.7 macrophage cells. Overexpression of SIK1 and SIK3, but not SIK2, inhibits LPS-induced NF-κB activity and impacts proinflammatory cytokine expression (Kim et al., 2013). Knockdown of SIK1 and SIK3 in Raw 264.7 cells leads to activation of NF-κB pathway (Kim et al., 2013). Another study reported that SIK3 deficiency exacerbates LPS-induced endotoxicity and is associated with higher expression of proinflammatory cytokines (Sanosaka et al., 2015). Thus, it remains to be understood for the isoform specific function of endogenous SIKs in regulating the proinflammatory cytokine expression (Jagannath et al., 2023).

In addition to their immunoregulatory roles in macrophages, SIKs also involve in zymosan-stimulated cytokine production in mouse bone marrow-derived dendritic cells (Sundberg et al., 2014), LPS or IL-1β induced cytokine modulation in human myeloid cells (Lombardi et al., 2016), and IL-33-stumulated cytokine secretion in mast cells (Darling et al., 2021). Furthermore, SIKs also modulate adaptive immunity by regulating T cell lineage commitment, differentiation, and survival via a MEF2C-dependent mechanism (Nefla et al., 2021; Canté-Barrett et al., 2022).

## Fibrosis

Fibrosis is a pathological process resulted from tissue injury or chronic inflammation (Henderson et al., 2020). It is characterized by the activation of fibroblasts and access accumulation of ECM proteins such as collagen and glycosaminoglycans (Henderson et al., 2020). Fibrosis is a hallmark feature of many cardiovascular disease. In pathological cardiac remodeling, myocardial fibrosis occurs as deposition of ECM proteins by activated fibroblast, which results in the expansion of cardiac interstitium and ultimately leads to systolic and diastolic dysfunction (Frangogiannis, 2021). Fibrogenic growth factors such as TGFβ, cytokines including TNF-α, IL-1, IL-6, IL-10, and IL-4, and neurohormonal pathways play essential roles in the pathogenesis of fibrosis (Frangogiannis, 2021).

**FIGURE 6:**
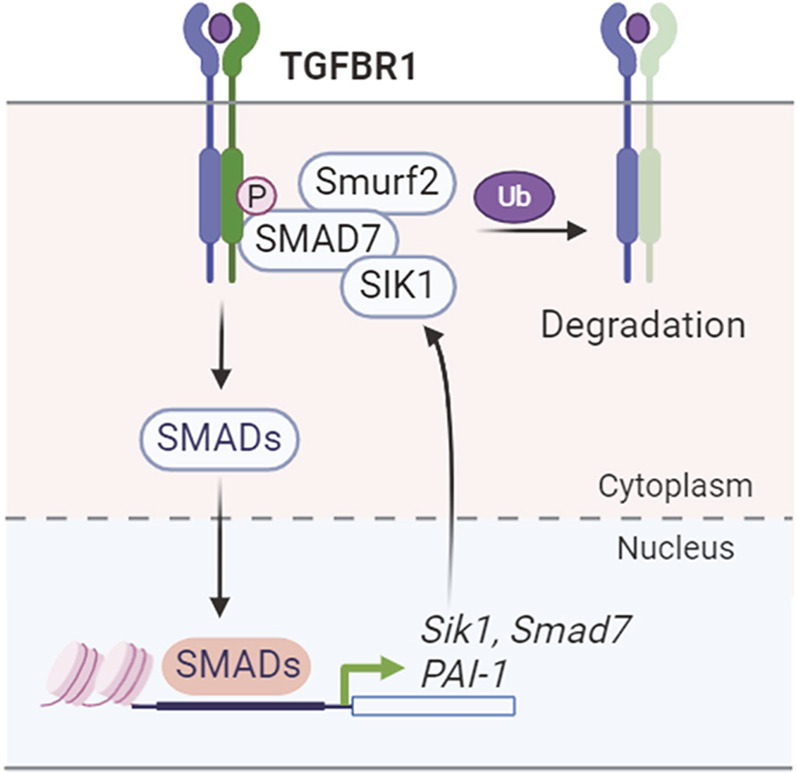
SIK1 regulation of TGFβ signaling.

SIK1 involves in a negative feedback regulation of the TGFβ signaling (Figure 6). TGFβ signals through type I and type II receptor serine/threonine kinases and intracellular Smad proteins to ultimately regulate the transcriptional program (Kowanetz et al., 2008). SIK1 is an inducible target of TGFβ pathway and acts to negatively regulate type I receptor kinase signaling (Kowanetz et al., 2008). Mechanistically, SIK1 forms a complex with Smad7 to downregulate the activated type I receptor TGFBR1 (ALK5) by increasing ubiquitination dependent receptor degradation (Kowanetz et al., 2008). Further study showed that TGFβ can direct regulate the transcription of *Sik1*, which involves in the binding of SMAD protein to the promoter of *Sik1* (Lönn et al., 2012). SIK1 forms a complex with the ubiquitin ligase Smurf2 to regulate type I receptor turnover (Lönn et al., 2012). *Sik1* deletion in mice induces collagen deposition and expression of genes related to ECM remodeling in the aorta (Bertorello et al., 2015). Furthermore, SIKs also regulate the TGFβ-targeted transcriptional gene program. Small molecule SIK inhibition or genetic SIK inactivation attenuates TGFβ-mediated transcription of PAI-1 (plasminogen activator inhibitor-1) and enhances cell apoptosis response (Hutchinson et al., 2020).

Pharmacological SIK inhibitors have demonstrated anti-fibrosis effects in mouse models. Earlier studies showed that dasatinib attenuates pulmonary fibrosis in bleomycin-exposed mice (Yilmaz et al., 2015) and in acute experimental silicosis (Cruz et al., 2016). However, whether the anti-fibrosis of dasatinib depends on SIKs is unclear. A recently study showed that SIK2 expression and activity is increased in fibrotic lung tissues and activated fibroblasts (Zou et al., 2022). A selective SIK2 inhibitor ARN-3236 restricts fibroblasts differentiation and ECM gene expression in human fetal lung fibroblasts and attenuates bleomycin-induced pulmonary fibrosis in mice (Zou et al., 2022), supporting as pathological role for SIK2 in pulmonary fibrosis.

## Metabolic syndrome

**FIGURE 7:**
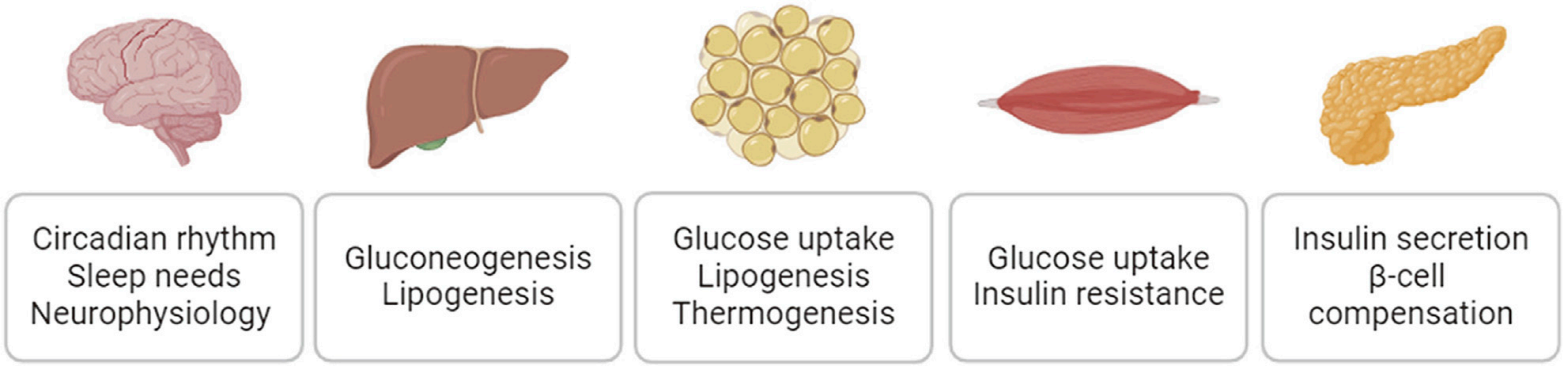
SIK regulation of glucose homeostasis and lipid metabolism.

As major risk factors for cardiovascular disease, diabetes, obesity, and metabolic syndrome negatively impact cardiovascular function (Strain and Paldánius, 2018; Powell-Wiley et al., 2021). The sodium/glucose cotransporter 2 (SGLT2) inhibitor empagliflozin and glucagon-like peptide-1 receptor (GLP1R) agonist semaglutide, medications originally developed to treat diabetes and/or obesity, have demonstrated clinically significant cardiovascular benefits (Cowie and Fisher, 2020; Sattar et al., 2021; Lincoff et al., 2023). It has been increasingly appreciated that targeting diabetes, obesity, and related metabolic dysfunction could be a disease modifying strategy for cardiovascular disease. SIKs are broadly expressed in the metabolic relevant tissues and play essential roles in mediating insulin action, maintaining glucose hemostasis, and controlling lipid metabolism (Figure 7). It has been appreciated that dysregulation of SIK function could underpin the pathophysiology of insulin resistance, dyslipidemia, and metabolic syndrome. In addition, SIKs are also expressed in the central nervous system and play essential roles in the regulation of circadian rhythm, sleep needs, and many other neurophysiological functions (Jagannath et al., 2023). Dysregulation of these neurophysiological processes could also have major impacts on systemic metabolic homeostasis and potentially contributes the cardiometabolic disease (Chellappa et al., 2019; Fagiani et al., 2022).

SIKs are critical regulators of hepatic gluconeogenesis. After feeding, insulin-stimulated FOXO (forkhead box O) phosphorylation by AKT leads to 14-3-3 binding and cytoplasmic restriction of FOXO, and thus inhibits the gluconeogenesis gene program (Wein et al., 2018). Under fasting condition, glucagon induces the PKA-mediated phosphorylation of CREB and recruitment of the p300 HAT and CREB binding protein (p300/CBP) to activate the gluconeogenesis gene expression (Wein et al., 2018). Glucagon action leads to PKA-mediated phosphorylation and inactivation of SIKs, which results in the dephosphorylation and nuclear translocation of CRTCs and class IIa HDACs. CRTCs bind to CREB and act as coactivator to stimulate the gluconeogenesis gene expression. Class IIa HDACs can recruit HDAC3 to the target gene promoters to regulate the acetylation of FOXO, which further activate the transcription of gluconeogenesis genes (Wein et al., 2018). In mouse models, liver specific ablation of LKB1, the upstream activating kinase for SIKs, leads to increased glucose production in hepatocytes. Pharmacological pan-SIK inhibition phenocopies LKB1 deficient hepatocytes, including the dephosphorylations in CRTC2/3 and HDAC4/5 and increased gluconeogenesis gene expression and glucose production (Foretz et al., 2010; Patel et al., 2014). However, deleting either *Sik1* or *Sik2* alone in the liver does not impact hepatic gluconeogenesis (Patel et al., 2014; Nixon et al., 2015), suggesting SIKs play redundant roles in hepatic gluconeogenesis.

SIKs also control hepatic lipogenesis. Hepatic lipogenesis gene expression is controlled by master transcription factors, including SREBP-1c (sterol regulatory element-binding protein 1c) and ChREBP (carbohydrate response element binding protein) (Wang et al., 2015). Hepatic *Sik1* knockdown increases lipogenic gene expression (Yoon et al., 2009). SIK1 suppresses hepatic lipogenesis gene expression by inhibitory phosphorylation of SREBP-1c at Ser329 (Yoon et al., 2009). *Sik2* knockdown in the liver also increases hepatic lipogenesis gene expression and steatosis (Bricambert et al., 2010). Mechanistically, SIK2 suppresses p300 HAT activity by inhibitory phosphorylation at Ser89, which in turn leads to decreased acetylation in ChREBP and reduced transcriptional activity of lipogenesis genes (Bricambert et al., 2010). Global *Sik3* deletion leads to neonatal death and skeletal malformations in survived adults (Uebi et al., 2012). The survived adult *Sik3* knockout mice show lipodystrophy phenotypes with altered cholesterol and bile acid metabolism in the liver (Uebi et al., 2012), indicating the involvement of SIK3 in lipid metabolism.

SIKs control a broad array of metabolic pathways in the adipose tissue. Among all the three SIK isoforms, SIK2 is the most abundant both in rodent and human adipocytes (Horike et al., 2003; Säll et al., 2016). SIK2 expression is robustly induced during adipocyte differentiation and its deletion leads to increased adipocyte differentiation in cell culture (Horike et al., 2003; Du et al., 2008; Gormand et al., 2014; Park et al., 2014). SIK2 can directly phosphorylates IRS-1 (insulin receptor substrate 1) *in vitro* and in cultured adipocytes, suggesting its involvement in insulin signaling (Horike et al., 2003). Insulin-stimulated AKT phosphorylation is reduced in the adipose tissue of global *Sik2* knockout mice (Park et al., 2014) and in human adipocytes treated with pan-SIK inhibitor (Säll et al., 2016). *SIK2* activity is increased in the adipose tissues from diabetic *db/db* mice (Du et al., 2008). However, *SIK2* mRNA expression and activity is downregulated in the adipose tissue of obese individuals (Säll et al., 2016). Global *Sik2* knockout mice show impaired glucose and insulin tolerance, hypertriglyceridemia, and adipose tissue dysfunction, such as increased lipolysis and macrophage infiltration and decreased GLUT4 and adiponectin expression (Park et al., 2014). The downstream molecular targets of SIK2 include HDAC4, CRTC2, and CRTC3 in adipocytes and support a role for SIK2 in regulating GLUT4 expression and adipocyte glucose uptake (Park et al., 2014; Henriksson et al., 2015). Lipogenesis gene expression is increased in the adipose tissue of *Sik2* knockout mice (Park et al., 2014). It has been shown that SIK2 inhibits the expression of lipogenic genes potentially by regulating SREBP1c in adipocytes (Du et al., 2008).

SIKs play redundant roles as suppressors of the adipocyte thermogenic program. The adipocyte thermogenic program is orchestrated by the β-adrenergic receptor signaling cascade to drive the transcriptional machinery for thermogenic gene expression (Shi and Collins, 2017; 2023). Combined deletion of *Sik1* and *Sik2*, but not deletion of *Sik1* or *Sik2* alone, increases the expression of thermogenic gene uncoupling protein 1 (*Ucp1*) in subcutaneous inguinal while adipose tissue (iWAT) (Wang et al., 2017). Knockdown of either *Sik1*, *Sik2*, or *Sik3* increases *Ucp1* expression in mouse brown adipocytes (Shi et al., 2023). In addition, treatment with a pan-SIK inhibitor YKL-05-099 in mice stimulates the thermogenic gene expression in iWAT (Shi et al., 2023). Furthermore, adipose tissue specific ablation of LKB1, the upstream kinase for SIK activation, promotes brown fat expansion and WAT browning (Shan et al., 2016; Wang et al., 2017). The adipose browning phenotypes of LKB1 adipose knockout mice are mediated by the action of SIK substrates CRTC3 and HDAC4 (Shan et al., 2016; Wang et al., 2017).

SIK1 involves in the CREB-dependent myogenic gene program. SIK1 is induced by the CREB transcription activity in skeletal myocytes and it promotes MEF2-dependent myogenic program by suppressing the class IIa HDAC activity (Berdeaux et al., 2007). SIK1 protein abundance is also dynamically controlled in response to cAMP stimulation during myogenesis (Stewart et al., 2012). *Sik1* depletion in myogenic precursors profoundly impairs MEF2 protein accumulation and myogenesis (Stewart et al., 2012). Overexpression of SIK1 reduces the muscle dystrophy caused by a dominant negative A-CREB mutation (Berdeaux et al., 2007). In adult mice, skeletal muscle *Sik1* expression can be induced by exercise training (Bruno et al., 2014), acute muscle injury (Stewart et al., 2011), and obesity (Horike et al., 2003; Nixon et al., 2015). Interestingly, global *Sik1* knockout mice are protected against high-fat diet induced insulin resistance (Nixon et al., 2015). *Sik1* deletion in skeletal muscle, but not liver or adipose tissue phenocopies the whole-body knockout mice, suggesting skeletal muscle *Sik1* contribute to diet-induced insulin resistance through a tissue autonomous mechanism (Nixon et al., 2015).

SIKs involve in the regulation of insulin secretion in pancreatic β-cells. *Sik1* deletion enhances glucose-stimulated insulin secretion in β-cells (Kim et al., 2015). Mechanistically, *Sik1* directly phosphorylates phosphodiesterase 4D (PDE4D) at Ser136/Ser141, which leads to reduction in cellular cAMP and attenuation of insulin secretion (Kim et al., 2015). In contrast, β-cell specific *Sik2* deletion reduces glucose-stimulated insulin secretion (Sakamaki et al., 2014). SIK2 phosphorylates CDK5R1 (p35) to induce its ubiquitination and proteasomal degradation, which leads to the suppression of CDK5 activity and activation of the voltage-dependent Ca^2+^ channels (VDCC) (Sakamaki et al., 2014). VDCC activation in turn induces Ca^2+^ influx and potentiates exocytosis and insulin vesicle release (Sakamaki et al., 2014). It was also proposed that SIK2 is involved in the β-cell compensation during hyperglycemia and loss of SIK2 contributes to β-cell failure and diabetes (Sakamaki et al., 2014).

## Development of SIK inhibitors

Small molecule SIK inhibitors (SIKi) have been developed as tools to probe the function of SIKs and have also shown therapeutic potential in various disease models, including osteoporosis, leukemia, inflammatory and fibrotic disease, and certain types of cancer (Wein et al., 2018; Sun et al., 2020; Darling and Cohen, 2021; António et al., 2022; Jagannath et al., 2023). HG-9-91-01 is the first widely used pan-SIKi for *in vitro* models as it can be rapidly degraded by mouse liver microsomes (Sundberg et al., 2016). HG-9-91-01 targets a hydrophobic pocket created by the presence of a small amino acid residue at the gatekeeper site in the SIK kinase domain (Clark et al., 2012). Mutation of threonine residue within the gatekeeper sites impairs the accessibility of inhibitor and confers resistance to HG-9-91-01 inhibition (Clark et al., 2012). Using HG-9-91-01 as a starting point, YKL-05-099 was development and it shows increased selectivity for SIKs and enhanced pharmacokinetic properties (Sundberg et al., 2016). A well-tolerated dose of YKL-05-099 *in vivo* achieves free serum concentrations above its IC_50_ for SIK2 inhibition for >16 h, making it the first pan-SIKi widely used for *in vivo* studies (Sundberg et al., 2016). YKL-05-099 has been shown to modulate immunoregulatory cytokine production (Sundberg et al., 2016), suppress acute myeloid leukemia progression (Tarumoto et al., 2020), and increase bone formation (Wein et al., 2016; Tang et al., 2021). Our group showed that YKL-05-099 stimulates adipocyte thermogenic gene expression and promotes adipose tissue browning *in vivo* (Shi et al., 2023), suggesting a role for SIKs in regulating energy hemostasis and their potential as therapeutic targets of obesity.

The three SIK isoforms are broadly expressed. Each SIK isoform could have both unique and redundant functions in a variety of physiological processes (Wein et al., 2018; Sun et al., 2020; Darling and Cohen, 2021; António et al., 2022; Jagannath et al., 2023). As such, both pan and isoform specific SIKi are desired to better approach these targets in specific disease contexts. Newer SIKi with better potency, specificity and oral bioavailability have been developed. GLPG3312 is a pan-SIKi developed by Galapagos NV that demonstrated both anti-inflammatory and immunoregulatory activities *in vitro* in human primary myeloid cells and *in vivo* in mouse models (Temal-Laib et al., 2023). JRD-SIK1/2i-4 is a selective SIK1/2 inhibitor that modulates innate immune activation and suppresses intestinal inflammation (Babbe et al., 2024). SK-124 is an orally available SIK2/3 inhibitor that stimulates bone formation without evidence of short-term toxicity in mice (Sato et al., 2022). GLPG3970 is a SIK2/3 inhibitor developed by Galapagos NV for the treatment of auto-immune and inflammatory diseases (Peixoto et al., 2024). Selective SIK2 inhibitors have been developed based on the binding pose of GLPG-3970 by the application of AlphaFold structures and generative models (Zhu et al., 2023).

ARN-3236 is an orally active and selective SIK2 inhibitor (Lombardi et al., 2016; Zhou et al., 2017). ARN-3236 induces an anti-inflammatory phenotype in human myeloid cells by modulating cytokine production after TLR agonist stimulation (Lombardi et al., 2016). ARN-3236 has also been shown to inhibit ovarian cancer growth and enhancing paclitaxel chemosensitivity in preclinical models (Zhou et al., 2017). Pterosin B is an indanone found in bracken fern (pteridium aquilinum) and a selective SIK3 inhibitor (Itoh et al., 2015; Yahara et al., 2016). It has been shown that pterosin B stimulates hepatic glucose production (Itoh et al., 2015) and prevents chondrocyte hypertrophy and osteoarthritis in mice (Yahara et al., 2016). However, our knowledge on the *in vivo* effects of pterosin B is very limited. More recently, OMX-0407 was reported as an orally available, single-digit nanomolar inhibitor of SIK3 (Hartl et al., 2022). OMX-0407 suppresses intratumoral NF-κB activity and inhibits tumor cell growth *in vivo* (Hartl et al., 2022). Inhibition of SIK1 activity is considered undesirable due to its essential role in blood pressure control and vascular remodeling (Bertorello et al., 2015). However, selective SIK1 inhibition might be beneficial in certain disease conditions (Kim et al., 2015; Hsu et al., 2020). Instead, phanginin A, a diterpenoid from the seeds of Caesalpinia sappan Linn (Yodsaoue et al., 2008), has been reported as a SIK1 activator to inhibit hepatic gluconeogenesis by increasing PDE4 activity and suppressing the cAMP signaling pathway (Liu et al., 2020).

## Conclusion remarks

Studies of genetic mouse models have demonstrated that SIKs are important regulators in the cardiovascular system and metabolic pathways (summarized in Table 1). It has been appreciated that SIKs could have both redundant and isoform-distinct functions in these pathophysiological processes. As such, the *in vivo* studies of genetic mouse models with SIK deletions might be challenging as efforts have to be made to delineate the redundant and distinct roles of each SIK protein in each tissue. These studies could be further complicated by the additional efforts required to identify the specific SIK substrate and their mechanisms of regulation in each physiological context. It has also been proposed that dysregulation of SIKs is associated with cardiometabolic disease. As mentioned above, a polymorphism in SIK1 gene encoding a G15S missense mutation is associated with lower blood pressure and reduced cardiac hypertrophy in humans (Popov et al., 2011; Bertorello et al., 2015). The expression and activity of SIK2 and SIK3 are downregulated in the adipose tissue of people with obesity and diabetes (Säll et al., 2016). Genetic variants in SIK genes also have been linked with alterations in blood lipid panels, suggesting the potential clinical relevance of SIK genes for dyslipidemia (Chasman et al., 2008; Teslovich et al., 2010; Braun et al., 2012; Willer and Mohlke, 2012; Ko et al., 2014; Karjalainen et al., 2020). For the translational development of SIKi, their cardiovascular and metabolic effects need to be considered and carefully evaluated. Understanding the cardiometabolic function of SIKs would not only offer new knowledge but also provide opportunity to better approach these targets and pathways for intervention and treatment of disease in the future.

**TABLE 1:** Cardiovascular and metabolic phenotypes of *Siks* deficient mouse models.

Mice	Disease models	Phenotypes	References
*Sik1* KO	High-salt intake	Higher blood pressure, increased vasoconstriction, sympathetic nerve system activity, and cardiac hypertrophy	Bertorello et al. (2015), Pires et al. (2019), Pires et al. (2021), António et al. (2022)
*Sik2* KO	High-salt intake	Prevents LV hypertrophy, normal blood pressure and plasma electrolytes homeostasis	Pires et al. (2021), Popov et al. (2014)
*Sik1/2* KO	High-salt intake	Higher blood pressure, prevents LV hypertrophy	António et al. (2022)
*Sik1* KO	Transverse aortic constriction	Improves LV systolic function, reduces cardiac hypertrophy and LV fibrosis	Hsu et al. (2020)
*Sik3* cmKO	Transverse aortic constriction	Exhibits cardiomegaly, increases cardiac hypertrophy and LV fibrosis	Hsu (2022)
*Sik2* KO	Myocardial infarction	Reduces hypertrophic response, infarct area and fibrosis	Liu et al. (2022)
*Sik1* smKD	Hypoxia-induced PAH	Increases RV hypertrophy and pulmonary arterial remodeling	Pu et al. (2022)
Pan-SIK inhibition	Femoral artery wire injury	Reduces neointima formation and vascular inflammation, attenuates vascular SMC proliferation	Cai et al. (2021)
*Sik1* KO	High fat diet	Normoglycemic on chow diet, prevents high-fat diet induced insulin resistance	Nixon et al. (2015)
*Sik2* KO	Chow diet	Hypertriglyceridemia, impaired glucose and insulin tolerance, adipose tissue dysfunction	Park et al. (2014)
*Sik3* KO	Chow diet	Postnatal lethality, skeletal malformation, dysregulation in glucose and lipid metabolism	Uebi et al. (2012)

Note: KO, knockout; KD, knockdown; cm, cardiomyocyte; sm, smooth muscle; LV, left ventricle; RV, right ventricle; PAH, Pulmonary arterial hypertension.

## Abbreviations

SIKs, salt-inducible kinases; SNF1, sucrose-nonfermenting 1; HDACs, histone deacetylases; CREB, cAMP responsive element binding protein; CRTCs, CREB-regulated transcriptional coactivators; MEF2, myocyte enhancer factor 2; LKB1, liver kinase B1; PKA, protein kinase A; AMPK, adenosine monophosphate-activated protein kinase; CaMK, Ca2+/calmodulin dependent kinase; PKD, protein kinase D; TGFβ, transforming growth factor β; PI3K, phosphoinositide 3-kinase; NKA, Na+/K+-ATPase; NCX, Na+/Ca2+ exchanger, VDCC, voltage-dependent Ca2+ channels; HAT, histone acetyltransferase; MI, myocardial infarction; PAH, pulmonary arterial hypertension; ECM, extracellular matrix; PAECs, pulmonary artery endothelial cells; PASMCs, pulmonary artery smooth muscle cells; SREBP-1c, sterol regulatory element-binding protein 1c; ChREBP, carbohydrate response element binding protein; MHC, myosin heavy chain; NF-κB, nuclear factor-κB; PME-1, phosphatase methylesterase-1, NRVMs, neonatal rat ventricular myocytes.
